# Impact Study: MK-0646 (Dalotuzumab), Insulin Growth Factor 1 Receptor Antibody Combined with Pemetrexed and Cisplatin in Stage IV Metastatic Non-squamous Lung Cancer

**DOI:** 10.3389/fonc.2015.00301

**Published:** 2016-01-13

**Authors:** Chao H. Huang, Stephen K. Williamson, Prakash Neupane, Sarah A. Taylor, Ace Allen, Nora J. Smart, Adelina M. Uypeckcuat, Sarah Spencer, Jo Wick, Holly Smith, Peter J. Van Veldhuizen, Karen Kelly

**Affiliations:** ^1^Kansas City Veterans Administration Medical Center, Kansas City, MO, USA; ^2^University of Kansas Cancer Center, Westwood, KS, USA; ^3^Department of Biostatistics, University of Kansas, Kansas City, KS, USA; ^4^University of California Davis Comprehensive Cancer Center, Sacramento, CA, USA

**Keywords:** dalotuzumab, non-squamous cell lung cancer, metastatic, IGF-1R, pemetrexed, treatment

## Abstract

**Background:**

Insulin-like growth factor 1 receptor (IGF-1R) regulates cell growth, proliferation, and apoptosis. Adenocarcinoma and never-smokers have a higher expression of IGF-1R, which is associated with worse overall survival. Dalotuzumab-MK0646 (D) is a humanized monoclonal antibody that targets IGF-1R. Pemetrexed (P) has higher activity in non-squamous lung cancer (NSQL). We initiated a randomized phase II trial to test the combination of P and Cisplatin (C) ± D in NSQL.

**Methods:**

Eligibility criteria were untreated NSQL stage IV, ECOG 0 or 1, measurable disease, adequate renal, hepatic and hematologic function, and no other intercurrent illness. P at 500 mg/m^2^ and C at 75 mg/m^2^ IV were given every 3 weeks. D was given at 10 mg/kg IV weekly on days 1, 8, and 15 of every 3-week cycle in the experimental group. The patients had a radiographic assessment after every two cycles and were treated for a maximum of six cycles if there was a response or stable disease. The primary objective of the study was to compare the clinical response rates of PC vs. PC + D.

**Results:**

From 1/2009 to 2/2011, the study accrued 26 subjects: 16 male and 10 female, with a median age of 59; 14 were treated with PC and 12 were treated with PC + D. We observed two partial responses (PR), seven stable disease (SD), three progressive disease (PD), and two not evaluable (NE) in the PC arm. In comparison, for the PC + D arm, there were three PR, four SD, four PD, and one NE. The hematologic toxicity was similar in both groups. There was higher incidence of hyperglycemia in the experimental group; four cases with grade 3 and one case with grade 4.

**Conclusion:**

PC + D had a similar response rate compared to PC, with a higher rate of hyperglycemia. Identification of responders using predictive markers would be key to continuing the study of D in NSQL.

**Trial Registration:**

NCT00799240, clinicaltrials.gov

## Introduction

**Figure 1:**
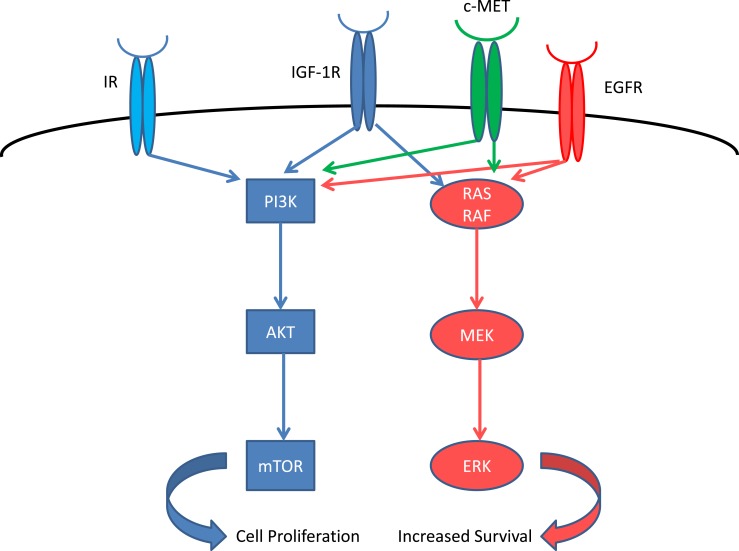
**IGF-1R system and related receptors**. IR, insulin receptor; EGFR, epidermal growth factor receptor.

Insulin-like growth factor 1 receptor (IGF-1R) is a tyrosine kinase receptor that regulates cell growth, proliferation, and apoptosis (1) (Figure 1). Increased IGF1 signaling results in increased proliferation and inhibition of apoptosis through RAF and PI3K pathways (2). Several types of cancer, including non-small cell lung cancer, express IGF-1R and its ligand. The serum levels of IGF are regulated by IGF-binding protein (IGFBP), which causes sequestration of IGF. Increased expression of IGFBP-3 is associated with improved outcome in resected stage I lung cancer (3). High expression of IGF-1R is associated with poor survival in surgically resected stage I adenocarcinoma which along with never-smokers had a higher expression of IGF-1R compared to squamous histology or smokers (4). Low IGF-1R expression was associated with significant improvement in survival in adenocarcinoma, while there was no correlation between IGF-1R expression and survival in patients with squamous histology. The use of an IGF-1R inhibitor is a potential new strategy in the treatment of lung cancer.

Figitumumab (F) was the first humanized monoclonal antibody targeting the IGF-1R studied in lung cancer. Initial phase II study showed promising results, especially in the squamous lung cancer histology (5). This agent was later studied in a large phase III trial but terminated early due to increased toxicity (6).

Dalotuzumab (D) or MK-0646 is a humanized monoclonal antibody specific to IGF-1R and does not bind insulin receptor (IR). It is an IgG1 isotype with potential to elicit an antibody-dependent cellular cytotoxicity (ADCC) response. The analysis of safety parameters of phase I studies demonstrated that D has been associated with thrombocytopenia, leukocytoclastic vasculitis, and hyperglycemia with no infusion reactions. Based on the phase I trial data, 10 mg/kg weekly is the recommended phase II dose. We proposed a randomized phase II trial to study the use of D in non-squamous lung cancer (NSQL) comparing pemetrexed (P)/cisplatin (C) vs. PC + D as first line therapy. We hypothesized that D could be synergistic with chemotherapy P + C in NSQL.

The study opened in January 2009 and it closed in February 2011 due to a decision from one of the sponsoring companies. We present data the 26 subjects enrolled in this study.

## Materials and Methods

The study was conducted under IND number 103120 and was reviewed and approved by the Institutional Review Board at the University of Kansas and Veteran’s Administration Medical Center Kansas City participating sites. The study was registered in the clinicaltrials.gov identifier NCT00799240.

The study included subjects 18 years or older who were able to give informed consent for treatment and laboratory exploratory studies. Patients needed an ECOG performance status of 0–1 with histologically or a cytological-proven newly diagnosed Stage IV (by AJCC seventh edition) advanced primary non-small cell bronchogenic lung cancer (non-squamous cell, including bronchoalveolar, adenocarcinoma, large cell carcinoma, or unspecified). If pleural effusion was clinically significant, thoracentesis was performed prior to initiation of therapy. Patients with symptomatic brain metastases were eligible after brain radiation and were neurologically stable, as well as off dexamethasone for at least 1 week prior to registration. Patients with asymptomatic brain metastasis were eligible if they did not require radiation and were neurologically stable without dexamethasone.

Measurable disease was documented by CT, MRI, X-ray, or physical exam, and assessed within 28 days before registration. Previous radiation or surgery was permitted if it was completed 1 and 4 weeks prior to registration. Patient must have recovered from all associated toxicities at the time of registration. Measurable disease must be present outside the area of surgical resection or radiation field; adequate bone marrow function which is defined by platelet count ≥100,000/mm^3^, hemoglobin ≥9 g/dl, leukocyte count ≥3,000/mm^3^, or absolute neutrophil count ≥1,500/mm^3^ and adequate hepatic function defined by bilirubin ≤1.5× upper normal limit, AST or ALT, and alkaline phosphatase all ≤3× institutional upper limit of normal (IULN) were required within 28 days prior to registration (except in presence of known hepatic metastasis, wherein AST or ALT may be up to 5× upper normal limit). Serum creatinine must be ≤ IULN with calculated or measured creatinine clearance must be ≥50 ml/mm using the Crockroft Gault formula. Women with childbearing potential required a negative serum pregnancy test; NSAIDS were to be discontinued 2 days before (5 days for long-acting NSAIDs), the day of, and 2 days following administration of P; folic acid, vitamin B12, and dexamethasone were administered according to protocol.

Subjects were excluded if they had prior systemic chemotherapy or biologic therapy for non-small cell lung cancer. If neoadjuvant or adjuvant therapy were given, they must be at least 1 year out from the last chemotherapy dose and fully recovered from all toxicities. Additional exclusion criteria were uncontrolled congestive heart failure, high blood pressure, unstable angina, or myocardial infarction within the prior year, serious cardiac arrhythmias requiring medication, serious uncontrolled active infection, acute hepatitis or known HIV, uncontrolled diabetes mellitus defined as a hemoglobin A1C ≥7. Patients with a prior history of severe allergy (grade 3 or 4) to human monoclonal antibody, concurrent use of human growth hormone or growth hormone inhibitors, and prior active malignancy in the past 2 years were also excluded. Persons of reproductive potential must have agreed to use two methods of effective contraception prior to, during, and for 4 weeks after study therapy.

## Statistical Considerations

The primary objective of the study was to compare the objective response rate between the two arms using RECIST criteria version 1. We estimated a target improvement from 30% in the control arm A using PC to 45% in the experimental arm B using PC + D. The secondary objective was to determine the progression-free survival (PFS) and overall survival (OS), the toxicity profile using CTC version 3, and exploratory laboratory analysis of IGF expression in the serum and tumor. PFS was defined as the duration of time from start of treatment to time of progression or death, whichever came first. Survival was determined from randomization to time of death due any cause. Kaplan–Meier curves and log rank tests were used to compare arms.

**Figure 2:**
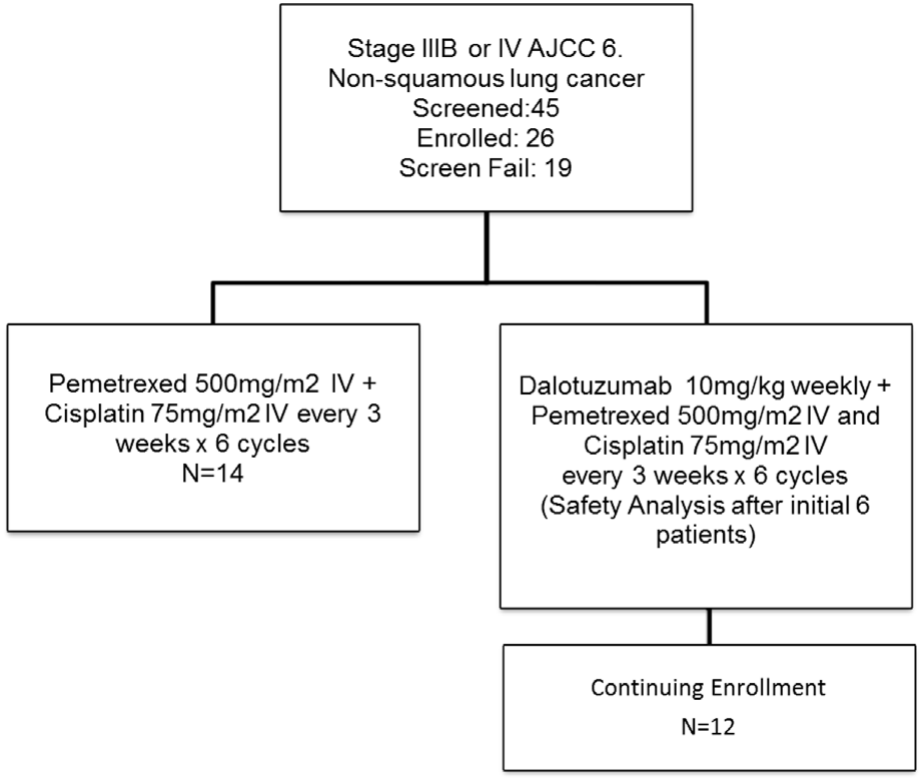
**Study schema**.

This was a randomized phase II study (Figure 2) designed to estimate the response rate based on multiple constraints. We wanted to estimate the response rate in each arm with a SE of no >10% as well as to estimate the margin of error for the difference in the response rates within 20%, with a 90% confidence interval. Three different scenarios were considered optimal, one-to-one randomization, and two-to-one randomization (experimental to control). Based on the expected response rates of 30% in standard of care and 45% in the experimental group, the one-to-one design was chosen. Thus, a total of 31 evaluable patients in each arm were required for this study. We planned to randomize a total of 68 subjects to allow for a 10% non-evaluable rate. Subjects were randomized upon entry into the study.

## Treatment

Arm A group received P at 500 mg/m^2^ IV every 21 days with C 75 mg/m^2^ IV every 21 days for six cycles with P infused first, followed by C. Arm B received PC with the same dose schedule as above with D at 10 mg/kg IV on days 1, 8, and 15 for every 21 days with PC infused first followed by D.

We gave premedication with vitamin supplementation consisting of folic acid 350–1,000 μg orally daily starting 1 week before P and continued during therapy; B12 1,000 μg IM starting 1 week before P then every 9 weeks during therapy and continuing until 3 weeks after completion of chemotherapy in both groups. Dexamethasone was given 4 mg bid/day the day before, the day of treatment, and the day after P. Patients also received standard pre- and post-hydration and antiemetic at the discretion of treating physician prior to C chemotherapy.

Arm B received additional premedication with benadryl 50 mg IV, acetaminophen 650 mg orally, and ranitidine 50 mg IV. As precaution, patients were observed for up to 1 h after the end of infusion of D. The observation area was supplied with resuscitation equipment and other agents (epinephrine, prednisone equivalents, etc.). Vital signs (blood pressure, heart rate, respiratory rate, and temperature) were checked prior to, midway, at the completion of D, and 1 h after D. The study utilized the NCI Common Terminology Criteria for Adverse Events (CTCAE) Version 3.0 for toxicity and Serious AE reporting. Dose modifications were performed according to pre-established toxicity criteria. Once a dose was reduced, it remained so for all subsequent cycles. If multiple toxicities occurred, dose modifications were based on the toxicity requiring the largest dose reduction.

We performed an interim toxicity analysis after six patients were enrolled in Arm B (PC + D), which was pre-specified in the protocol. There were no observed excessive grade 3 or 4 toxicities and the study continued to enroll additional subjects.

Patients were removed from protocol treatment if they had progression of disease as defined by RECIST 3.0; symptomatic deterioration; unacceptable toxicity, chemotherapy treatment delay for >3 weeks from the scheduled treatment date for any reason; D treatment delay >4 weeks from the scheduled treatment date for any reason except hyperglycemia; or by patient choice. After completion of active therapy, patients were followed every 8 weeks for 18 months after registration or until disease progression or death (whichever came first).

## Results

The study accrued 26 subjects, and it was stopped due a sponsor’s decision. We had 19 patients that did not enter in the trial due screen failure due a variety of reasons (7 patients declined to participate, 2 had recent surgery and did not recover, 1 due to physician decision, 1 had EGFR mutation positive and went to have treatment with EGFR TKI, 1 hospitalized due disease, 1 had insurance disapproval to participate in clinical trial, 1 due study termination) with 5 not meeting study criteria (2 had performance status >1, 1 due to creatinine clearance bellow 50 ml/min, 1 had squamous histology on pathology review, and 1 did not have stage IV on PET scan).

There were 10 females and 16 males, with a median age of 59 years old (range 41–83). Four had malignant pleural effusion and 22 had distant metastatic disease. We accrued 14 in arm A (PC) and 12 in arm B (PC + D). Dose reduction occurred in three patients in arm A: two patients that had dose reduction of C (given at 37 mg/m^2^) due to renal dysfunction and one patient had dose reduction of P (given at 375 mg/m^2^) and C. Dose reduction also occurred in three patients in arm B: two patients that had dose reduction of C due renal dysfunction and one patient had dose reduction of C and Dalotuzumab (given at 6 mg/kg) at cycle 6 day 15 due hearing toxicity. The number of cycles given in arm A was six cycles (6); five cycles (1); four cycles (2); three cycles (1); two cycles (2); and one cycle (1). Reason for discontinuation before six cycles were six due to progression of disease, one due renal failure and decrease in performance status, and one patient was lost to follow-up. The number of cycles given in arm B was six cycles (7); five cycles (1); two cycles (3); and one cycle (1). Reason for discontinuation before six cycles was four patients due progression of disease and one due renal failure (Table 1). The AE events are listed in Table 2. The hematologic toxicities were comparable between both groups. As would be predicted from an IGF-1R inhibitor, there was a higher incidence of hyperglyecemia (three grade 3 and one grade 4) in arm B vs. none in arm A. There was one toxicity-related death in arm A after complications of chemotherapy due to sepsis.

**Table 1:** **Patient characteristics**.

Number of patients	*N* = 26	
Gender	10 Female:16 male	
Median age	59 (range 41–83)	
Disease sites	Malignant effusion (4) distant metastases (22)	

**Treatment**	**Pemetrexed/cisplatin (PC) *N* = 14**	**Dalotuzumab with PC *N* = 12**

ECOG performance status	0 (4) 1 (10)	0 (4) 1 (8)
Median age	61	58
Gender	8 Male:6 female	8 Male:4 female
Stage IV	Malignant pleural effusion (3)	Malignant effusion (1)
	Distant disease (11)	Distant disease (11)
Sites of disease	Bone (7)	Bone (5)
	Brain (3)	Brain (4)
	Adrenal (4)	Adrenal (3)
	Liver (3)	Contralateral lung (3)
	Extra-thoracic node (2)	Extra-thoracic node (2)
	Chest wall (1)	Malignant pleural effusion (1)
	Malignant pleural effusion (4)	
No. of cycles	6 Cycles (6)	6 Cycles (7)
	5 Cycles (1)	5 Cycles (1)
	4 Cycles (2)	2 Cycles (3)
	3 Cycles (1)	1 Cycle (1)
	2 Cycles (2)	
	1 Cycle (1)	

**Table 2:** **Toxicities related to therapy**.

	Pemetrexed/cisplatin Arm A		Dalotuzumab with pemetrexed/cisplatin Arm B	
	Hematologic	Non-hematologic	Hematologic	Non-hematologic
Grade 1	Platelet (1); INR (1); leukocyte (1); hemoglobin (1)	5 Most common: nausea (13); hypokalemia (7); constipation (6); creatinine (6); fever (3)	Hemoglobin (7); leukocyte (6); neutrophil (5)	5 Most common: nausea (7); diarrhea (5); fatigue (5); hyperglycemia (5); anorexia (4)
Grade 2	Hemoglobin (4); leukocyte (4); neutrophil (2); platelet (2)	Fatigue (5); tinnitus (4); diarrhea (4); vomiting (4); nausea (4)	Leukocyte (6); lymphopenia (4); neutrophil (3); hemoglobin (2)	Fatigue (7); hyperglycemia (5); tinnitus (3); dehydration (2); hypotension (2)
Grade 3	Febrile neutropenia (2); leukopenia (1); hemoglobin (1); neutrophil (1); platelet (2)	1 Each: hyponatremia; dysphagia; urinary tract infection; fatigue; hypoalbuminemia, metabolic laboratory; esophageal stenosis; hypoxia, muscle weakness; dyspnea (2)	Hemoglobin (3); neutrophils (3); lymphopenia (1)	Hyperglycemia (4); 1 each: hearing; pain extremity; muscle weakness; hypokalemia; nausea
Grade 4	Hemoglobin (1); platelet (2)	Hypoxia (1); sepsis (1)		Hyperglycemia (1)

**Figure 3:**
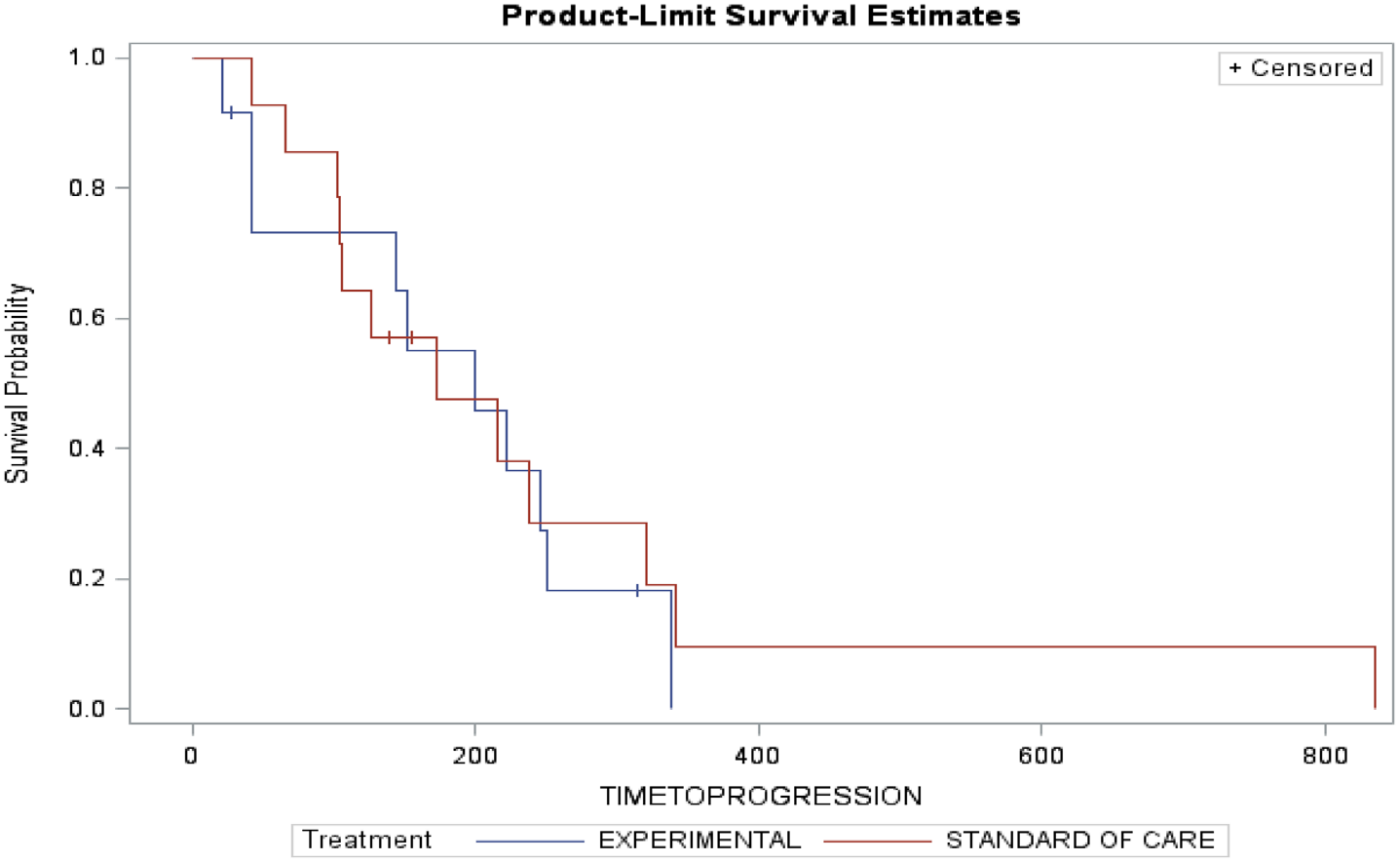
**Time to progression**.

**Figure 4:**
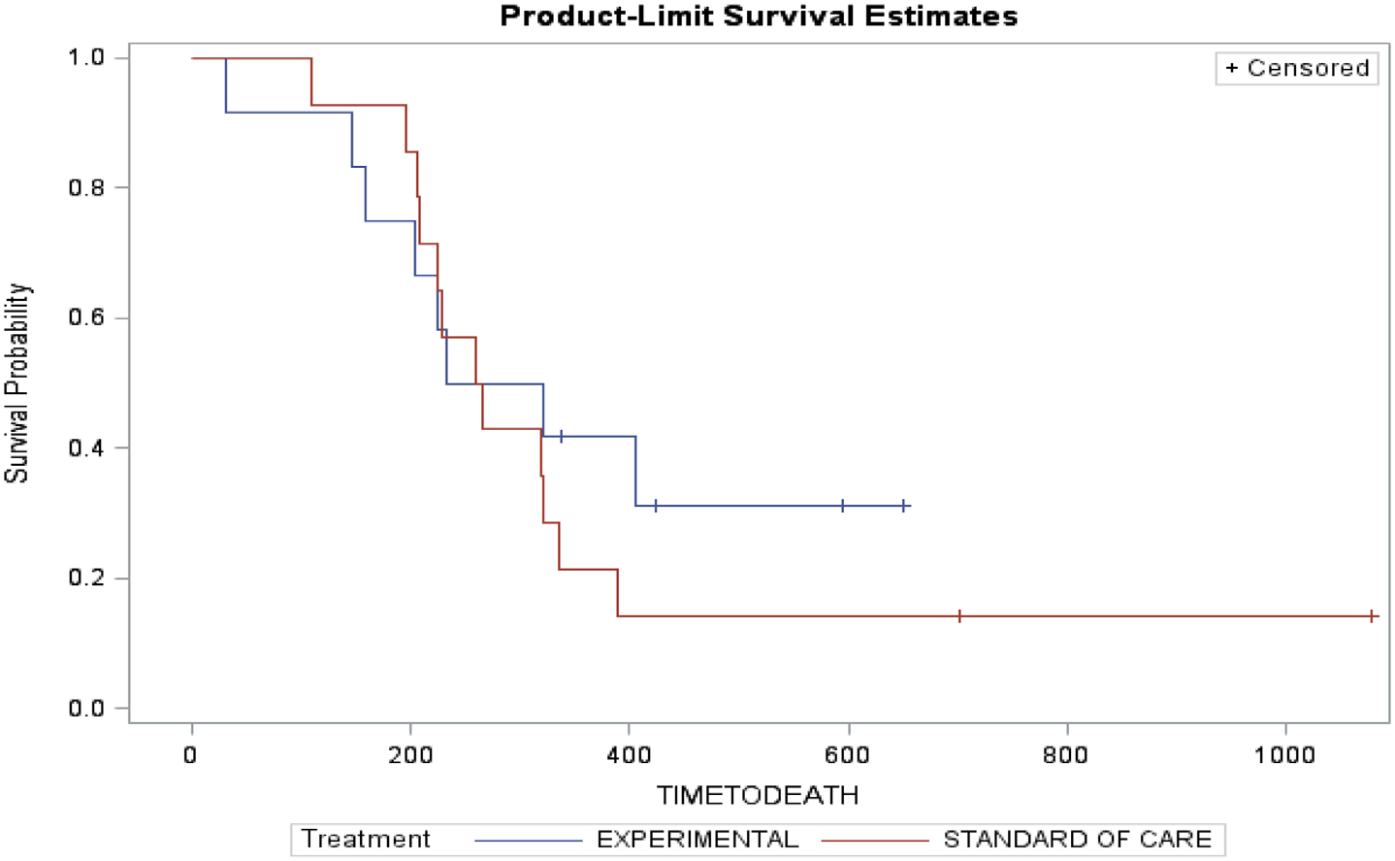
**Overall survival**.

Arm A (PC) had two partial responses (PR) (14%), one stable disease (SD) and three disease progression with two patients not evaluable (NE) (one due to complications from chemotherapy and one lost to follow-up). The median time to progression was 173 days and the median survival was 262 days. Arm B (PC + D) had three patients with PR (25%), four SD, four disease progression, and one NE. The median time to progression was 199 days and median survival was 276.5 days (Table 3). The figures for time to progression, OS, and an example of the response obtained, are depicted in Figures 3 and 4, respectively. Incomplete accrual limited statistical analysis.

**Table 3:** **Results**.

Arm A	Arm B
Pemetrexed/cisplatin	Dalotuzumab with pemetrexed/cisplatin
PR 2	PR 3
SD 7	SD 4
PD 3	PD 4
NE 2	NE 1
**Median time to progression**	**Median time to progression**
173 days	199 days
**Median survival**	**Median survival**
262 days	276.5 days

*PR, partial response; SD, stable disease; PD, progression of disease; NE, not evaluable*.

## Discussion

The first clinical experience using an IGF-1R monoclonal antibody was with F. Preclinical studies showed that it has activity as a single agent and it was synergistic when combined with chemotherapy (7, 8). A phase I study in 24 patients showed no toxicities greater than grade 3 with clinical activity, 3 SD, and 7 minor tumor reduction (9). A phase II study combining F with paclitaxel/carboplatin (TCb + F) as first line treatment of advanced non-small lung cancer had 2:1 randomization with 73 patients. It compared TCb + F for six cycles followed by maintenance with F vs. TCb alone with potential for crossover if progression on or no response with TCb alone. The study showed a response rate of 46% in the TCb + F arm vs. 32% in the TCb arm. Subgroup analysis showed a 71% response in patients with squamous cell histology. There was evidence of single-agent activity in one patient who progressed on TCb, then crossed over to F alone with stabilization of disease (5). Hyperglycemia (12%) and dehydration (58%) all grades were the most common side effects. A subsequent phase III trial using TCb with or without F in non-small cell lung cancer was suspended by the Data Safety Monitoring Board in September 2009 due to increased grade 5 toxicity in the TCb + F arm (6). Grade 5 AE occurred in 13% of patients in the TCb + F compared with 10% in TCb arm. Treatment-related grade 5 AE occurred in 5% of TCb + F and 1% in the TCb arm. The response rate, median PFS, and median OS for the TCb + F arm and TCb arm were similar, 33 vs. 35%; 4.7 vs. 4.6 m, and 8.6 vs. 9.8 m, respectively. Subsequent interim analysis showed that addition of F with TCb would not to meet the primary endpoint of improving OS compared with TCb. Since the presentation of this phase III trial in the ASCO 2010 annual meeting, several companies halted the development of this class of agent in lung cancer. The publication of the phase II trial with F was later retracted by the authors in October 2012 (10). The reason for retraction was due to non-adherence to the RECIST recommendation for follow-up CT scan to confirm responses discovered during routine study close-out review. The revised response rate was 37.4% in the TCb + F arm and 27.5% in the TCb arm. The response in the squamous histology was 43.8%. The median PFS was 4.3 m, 4.4 m for F at 10 mg/kg, and 4.5 m at 20 mg/kg, *p* = 0.816.

In our study with a limited number of patients treated with D, we observed similar response rates, PFS, and OS in the group receiving PC + D and PC. There was difference in the toxicity regarding hyperglycemia in patients treated with D most likely due to class effect. These results are not definitive since the study did not complete accrual to allow proper statistical analysis. We did not observe a higher grade 5 toxicity in the experimental arm, implying that there may be a difference of these antibodies, chemotherapy used, or lack of maintenance therapy with D. Unfortunately, we cannot clarify these questions since IGF-1R class of drug is no longer in the development of lung cancer.

If we were to resurrect the study of this agent, future research should be guided by a biomarker to help in patient selection. Serum levels of free IGF-1 might be such a biomarker. The phase III trial using F showed that patients with lower levels of baseline IGF-1(<120 ng/ml) had increased incidence of grade 5 AE vs. higher levels of IGF-1, 56% and 35%, respectively, with HR of 1.37 in patients with low baseline IGF-1 treated with TCb + F. The median OS was better in patients with higher levels of IGF-1 compared with lower levels of IGF-1 treated with TCb and F, 10.4 and 7 m, respectively. A similar effect of IGF-1 levels and outcomes were demonstrated in a study by Hixon et al. The median PFS was longer in patients with high free IGF-1 when they received TCb + F (20 mg/kg) vs. TCb, 6.53 and 2.73 months, respectively (*p* = 0.001) (11). Altogether, this information indicate that high IGF-1 level may help us select the population that will benefit from IGF-1R-based therapy with lower toxicity. Exactly how the high levels of IGF-1 result in lower toxicity is not clear.

Another area of research should be in mechanism of resistance and in formulation of strategies to overcome this resistance. The IGF-1R pathway activates both the PI3K/AKT/mTOR and RAS pathways in parallel (12). Furthermore, the homolog IR (13) as well as the cMET (14) and EGFR (15) pathways could also be involved in the resistance by simultaneous over-activation, in order to evade the blockade of IGF-1R. Mutation in these receptors and downstream protein during the course of therapy could lead to resistance to IGF-1R-targeted agents and render them ineffective. The complexity of these pathways is tremendous and further research using genomic/exome sequencing could determine the predominant mechanism of resistance, and assist us in the selection of agents to be used in combination with IGF-1R-targeted agent in order to circumvent resistance and characterize the patient population that will benefit from this therapy.

## Conclusion

This report is the first clinical report using D in non-squamous cell lung cancer in combination with PC. The study showed similar response rate, PFS, and OS in both groups; however, this results lack statistical significance since the study did not complete accrual. We did not observe significant increased hematologic or grade 5 toxicity in this limited group of patients treated with PC + D. We observed increased frequency of grade 3 hyperglycemia associated with D which is a class effect. The use of IGF-1R-targeted therapy is no longer under investigation in lung cancer. It is possible that patients with high IGF-1 levels could benefit from IGF-1R directed agents with lower toxicity. Future research should be directed toward defining response biomarker, mechanism of resistance, and patient selection that would benefit from IGF-1R pathway inhibition.

## Author Contributions

CH: study design, study conduct, patient enrollment, data review, data analysis, writing and review of manuscript. SW: study conduct, patient enrollment, data review, writing and review of manuscript. PN: study conduct, patient enrollment, data review, writing and review of manuscript. ST: study conduct, patient enrolment, data review, writing and review of manuscript. AA: study conduct, patient enrollment, data review, writing and review of manuscript. NS: study conduct, patient enrollment, data entry, writing and review of manuscript. AU: study conduct, patient enrollment, data entry, writing and review of manuscript. SS: study conduct, patient enrollment, data entry, writing and review of manuscript. JW: data review, data analysis, writing and review of manuscript. HS: data review, writing and review of manuscript. PV: data review, writing and review of manuscript. KK: study design, study conduct, patient enrollment, data review, writing and review of manuscript.

## Conflict of Interest Statement

Research Funding from Merck and Pemetrexed chemotherapy supplied by Eli Lilly.
